# Understanding the Role of Sensor Optimisation in Complex Systems

**DOI:** 10.3390/s23187819

**Published:** 2023-09-12

**Authors:** Burak Suslu, Fakhre Ali, Ian K. Jennions

**Affiliations:** Integrated Vehicle Health Management Centre, School of Aerospace, Transport and Manufacturing, Cranfield University, Bedfordshire MK43 0AL, UK; f.ali@cranfield.ac.uk (F.A.); i.jennions@cranfield.ac.uk (I.K.J.)

**Keywords:** sensor optimisation, complex systems, cost function, IVHM, aircraft health management

## Abstract

Complex systems involve monitoring, assessing, and predicting the health of various systems within an integrated vehicle health management (IVHM) system or a larger system. Health management applications rely on sensors that generate useful information about the health condition of the assets; thus, optimising the sensor network quality while considering specific constraints is the first step in assessing the condition of assets. The optimisation problem in sensor networks involves considering trade-offs between different performance metrics. This review paper provides a comprehensive guideline for practitioners in the field of sensor optimisation for complex systems. It introduces versatile multi-perspective cost functions for different aspects of sensor optimisation, including selection, placement, data processing and operation. A taxonomy and concept map of the field are defined as valuable navigation tools in this vast field. Optimisation techniques and quantification approaches of the cost functions are discussed, emphasising their adaptability to tailor to specific application requirements. As a pioneering contribution, all the relevant literature is gathered and classified here to further improve the understanding of optimal sensor networks from an information-gain perspective.

## 1. Introduction

### 1.1. Background to the Literature

Health management applications are critical for ensuring the reliable and effective operation of mission and safety-critical complex engineering systems, such as spacecraft, submarines, aircraft, and industrial plants. These applications rely on sensors that generate useful information about the health condition of the assets; thus, optimising the sensor network quality while considering specific constraints is the first step in assessing the condition of assets. This is particularly important for systems that operate in harsh environments, where the accuracy and reliability of sensor data are essential for effective maintenance and operation.

Integrated vehicle health management (IVHM) involves monitoring, assessing, and predicting the health of various systems within a vehicle or a larger system. This approach aims to provide a comprehensive understanding of the system’s health, by integrating data from various sensors and other sources and analysing it using advanced algorithms and analytical techniques to support condition-based maintenance (CBM) [1]. In recent years, there has been a growing interest in sensor optimisation techniques for IVHM, which concern optimising sensor placement, selection, and data processing to improve the accuracy and reliability of health monitoring systems.

Sensing technology is an essential tool for understanding and interacting with the physical world; by providing accurate and timely data, sensors allow us to make decisions and reason about the physical phenomena in an environment. IVHM systems build on this to provide accurate and comprehensive information about the state of the system with an optimal sensor suite that contributes to the increased availability of the system and effective reasoning over performance and maintenance actions. The sensor optimisation problem in complex systems involves finding the most effective combination of sensor types and locations that will provide the most accurate and comprehensive monitoring of a system.

In order to optimise sensor selection for IVHM or complex systems, various factors must be considered, including the cost, weight, size, and power consumption of the sensors. These factors can impact the feasibility and practicality of using certain types of sensors and can affect the overall performance and accuracy of the IVHM system. Historically, these objectives, from an engineering perspective, are viewed as the sensor optimisation problem; however, this study takes a broader view, including the information gained from the sensor suite.

Additionally, the optimisation problem in sensor selection also involves considering trade-offs between different performance metrics. For example, from information theory, increasing the number of sensors may improve the accuracy of the IVHM system but may also increase the weight, cost and computational complexity of the system. Balancing these trade-offs requires careful consideration of the specific requirements and constraints of the application, as well as a deep understanding of the underlying physics and dynamics of the system being monitored. Another important factor in sensor selection is the type of data that is required for fault detection and diagnosis. Different types of sensors may be better suited for monitoring different aspects of a vehicle’s health, such as temperature, pressure, vibration, or fluid levels. By selecting the right combination of sensors, IVHM systems can ensure that they are able to detect potential faults and provide accurate and timely alerts to maintenance personnel.

This review intends to provide a comprehensive overview of the existing literature on sensor optimisation for complex systems. It will explore various optimisation techniques used in different domains and highlight the key findings and limitations of each approach. Additionally, the review will identify areas for future research and discuss the potential benefits of sensor optimisation for IVHM systems.

### 1.2. Problem Statement

The use of IVHM systems in aircraft operation has become increasingly important for ensuring safety, increasing operational efficiency, and reducing downtime and maintenance costs. However, the effectiveness of these systems relies on the identification of optimal sensors and their locations. Sensors are used to translate physical phenomena into digital form, and the optimisation of these sensors can be performed in three different ways; physical, system and algorithmic. The first way is the improvement in the quality of the sensing technology itself, the second is location and quantity combined to obtain better information quality, and the last involves improving processing techniques.

The number of heterogenous parameters in flight data, collected from different types of sensors in the aircraft, is increasing due to high safety requirements, incident and accident investigation, maintenance, and diagnostic purposes. Analysing the entire sensor dataset, including up to thousands of parameters in modern aircraft, is neither practical nor computationally manageable for onboard diagnostic purposes. The data types range from Boolean data, to control system BITE codes, to high-frequency vibration or acoustic data. The analysis of the latter, which relies on defining which features in the data have more importance in terms of clear condition indicators, is an open research area.

The optimal sensor-suite identification problem in IVHM systems involves determining the sensor suite that will provide the most comprehensive and accurate monitoring of the aircraft’s health situation. This requires consideration of various factors, such as the type of data required for fault detection and diagnosis, the cost and weight of sensors, and the power consumption. While there has been significant research in this area, there remains a need for further investigation of the system-level optimisation of sensor suites for IVHM systems.

There are numerous research papers published on aircraft component and subsystem-level sensor optimisation, yet only a few papers consider system-level condition monitoring in aircraft. There is a need for further investigation into the use of advanced optimisation techniques and the integration of multiple subsystems for more comprehensive health management. Different complex engineering systems that utilise sensor optimisation techniques will be investigated to improve the current research in the IVHM domain.

**Figure 1:**
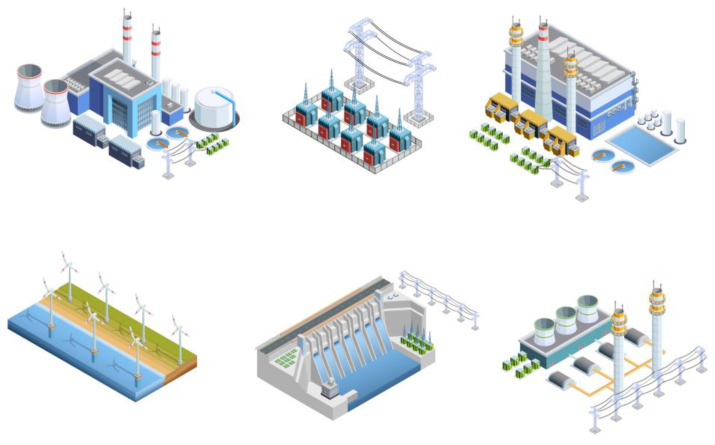
Some examples of Complex Systems.

Also, other complex system applications that benefit significantly from sensor optimisation are wind turbines, power generation plants, railways, satellites, etc. Figure 1 shows some examples of complex systems. Kulkarni et al. discussed an outline for a sensor-selection framework specifically designed for diagnostic applications, and they used wind turbines in their experiment [2].

**Figure 2:**
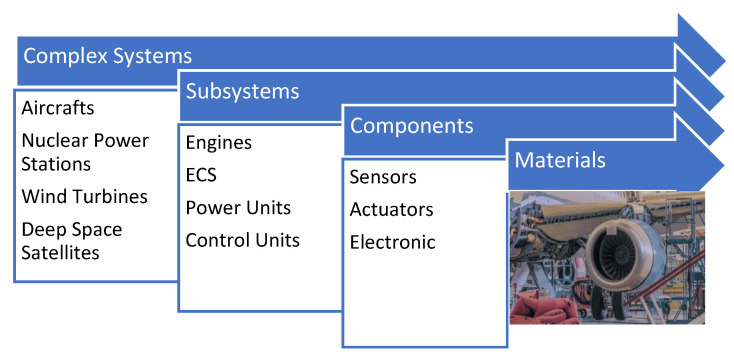
Direction and Examples of the Complex Systems’ Themes.

Defining a step-by-step methodology to rank/identify aircraft sensors for system-level condition monitoring and the optimisation of the sensors for onboard/online diagnosis where several domain-specific objective functions and constraints occur is addressed in this study. Additionally, faulty sensor detection, which comprises most of the faults in the aviation industry, is mainly neglected in the optimal sensor-suite selection research. This issue will also be considered for integration in the sensor-suite selection part. Figure 2 outlines the direction and examples of complex system’s themes.

The motivation for further research in sensor optimisation for IVHM systems is driven by the potential benefits of more accurate and reliable onboard diagnosis for health monitoring systems. This could lead to improved safety and availability as well as efficient CBM. Additionally, advances in sensor technologies and optimisation techniques present new opportunities for many different fields. The research will also identify areas for future investigations and highlight the potential benefits of a detailed sensor optimisation strategy in IVHM, providing guidance for researchers and practitioners in the field.

### 1.3. Scope of the Study

This research is focused on exploring the existing literature on sensor optimisation for: placement, selection, operational applications, and data processing techniques that are used to improve diagnostic information.

A systematic and thorough approach to sensor selection must be carefully designed to ensure the data collected are relevant and useful in the target application. The following questions need to be considered in the design phase:

- What is the specific application for which the sensor data will be used? This will help guide the selection of appropriate sensors and data collection methods.
- What are the key variables or parameters that need to be measured to achieve the desired outcome? For example, if the goal is to monitor the performance of a machine, collecting data on factors such as temperature, vibration, and power consumption is needed.
- What is the required level of accuracy for the data? This will help determine the appropriate resolution and precision for the sensors.
- What is the frequency at which data need to be collected? This will impact the selection of sensors and the required data storage and processing capabilities.
- What are the environmental factors that may affect sensor performance, such as temperature, humidity, and electromagnetic interference? This will help guide the selection of sensors that are capable of operating reliably under the specific conditions of the application.

By answering these elementary questions and conducting a thorough analysis of the target system and the specific applications, a framework can be designed to ensure that the selected sensors are appropriate for the task at hand and that the resulting data are accurate and useful for analysis and decision-making.

## 2. Structuring the Review

### 2.1. Methodology of the Review

To conduct a literature review on sensor optimisation in the complex system domain, electronic databases, including scientific journals, conference proceedings, and relevant books, were searched by using keywords related to the topic. The articles and books were then screened for their relevance to the research gaps, and only those that met the inclusion criteria were selected for review.

The inclusion criteria for this review are studies that examine sensor optimisation in complex systems to improve diagnostic information quality, particularly those used in the aviation domain. The physical improvement of the sensing technology itself and the structural health management (SHM) field are beyond the scope of this research. Some SHM examples are included in order to demonstrate the use of the proposed method.

In this review, our methodology encompasses a systematic literature selection process, including database searches and well-defined inclusion criteria, to gather pertinent sensor optimisation research. We meticulously extract and categorise data from selected sources, enabling a comparative analysis of sensor optimisation techniques, application domains, and future trends. Our review methodology culminates in a concise summary of key findings, emphasising the practical implications of sensor optimisation across diverse domains. This structured approach ensures methodological rigour and contributes to advancing the understanding of sensor optimisation techniques within complex systems.

Table 1 shows search results from the Scopus data. Search terms included various combinations of keywords.

Without any restrictions, a search on sensor and optimisation yielded 75,169 articles that are based on their titles, abstracts and keywords. When the other identified keywords of the study were added, this number dropped, as shown in the table, until a result with only 85 articles was found. When a stricter grouping was explored (by using quotation marks), it resulted in nine articles that consider different complex systems.

After analysing the relevance and contribution of each article to the field, the information found was synthesised in order to categorise the articles into different themes and analyse the main findings and trends. Identified associated paper references were also searched to expand the knowledge that was gathered over the field, and additional studies that met the inclusion criteria were included.

### 2.2. Taxonomy

A taxonomy that was developed through a comprehensive analysis of the results obtained from a Scopus search, as well as relevant books and papers, is presented in Table 1. The primary objective of this taxonomy is to classify and organise the various approaches used to optimise sensor suites for complex systems. The taxonomy’s classification criteria are based on the key domains and techniques employed in the process of sensor-suite optimisation.

At the highest level of categorisation, the taxonomy consists of several main branches, each representing a significant area or approach used in sensor optimisation. These branches serve as the fundamental divisions, providing an overarching structure to the taxonomy. Subsequently, within each main branch, further divisions and subcategories are established by posing fundamental investigative questions. These questions are designed to explore and dissect the various aspects and intricacies of optimising sensor suites for complex systems comprehensively.

**Figure 3:**
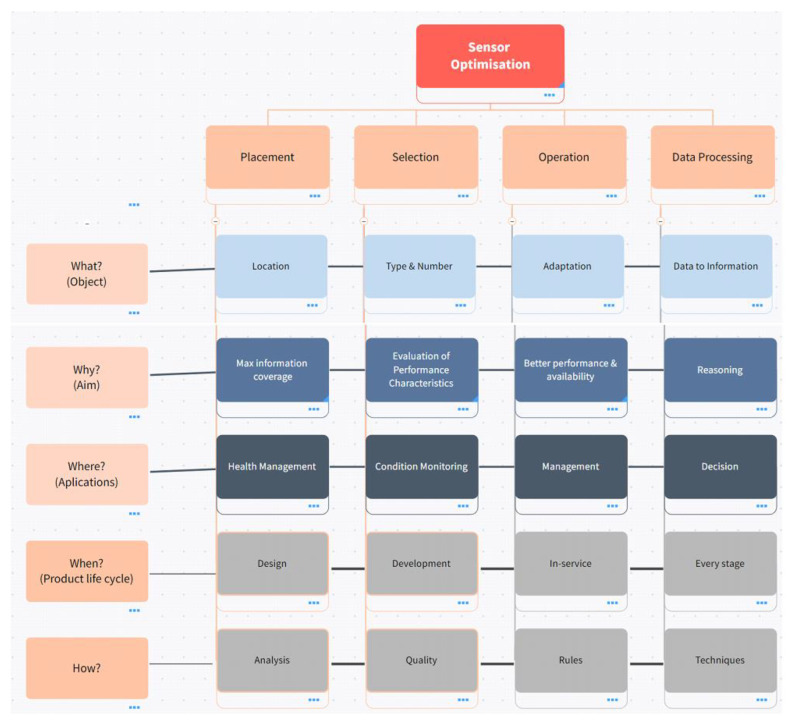
Taxonomy of Sensor Optimisation.

By employing this matrix organisation table, the taxonomy facilitates a systematic and methodical examination of the field, enabling researchers and practitioners to navigate through the vast array of sensor optimisation methods and concepts effectively Figure 3 represents the taxonomy of sensor optimisation.

This approach not only provides a comprehensive overview of the field but also offers actionable insights and recommendations for enhancing sensor performance and capabilities within complex systems. The initial taxonomy serves as an inclusive framework, laying the groundwork for subsequent sections to delve into sensor improvement areas. By leveraging the strengths of approaches, this paper can deliver a well-rounded exploration of sensor optimisation while offering targeted recommendations for improvement wherever appropriate.

**Figure 4:**
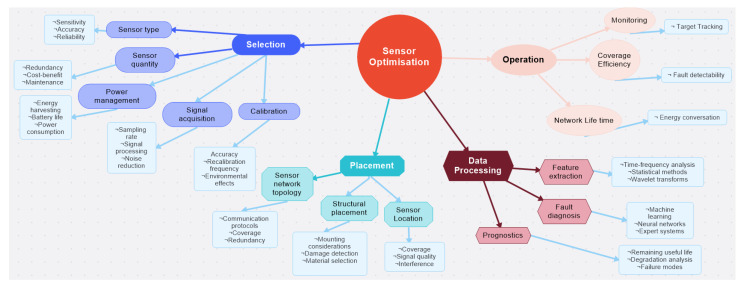
Conceptional Map of the Literature.

Figure 4 presents a concept map that has been thoughtfully organised to align with the key themes derived from the comprehensive literature review. Each major title in the concept map corresponds to a significant area of investigation, facilitating a clear understanding of the interrelationships and connections between the various concepts and topics explored in the reviewed literature.

The concept map visually organises the main themes from the literature review, providing a coherent and interconnected representation of the key concepts and considerations in sensor optimisation within complex systems. These brief descriptions offer readers a clear roadmap for further exploration and understanding of the topics presented in Figure 4. Brief descriptions of the main branches are given in the following paragraphs.

Selection: The type and number of sensors employed in the system must all be considered throughout the selection process. Making sure the chosen sensors are adequate for the task and deliver accurate and reliable data is the major goal of sensor selection. To this end, there are several factors that need to be assessed accordingly, like power management, signal acquisition and calibration. Choosing a certain sensor-type consideration of sensitivity, accuracy, or dependability is imperative. Sensor quantity entails choosing the right number of sensors to balance redundancy, information gain, and cost.

Placement: The placement of sensors requires taking into account their physical location as well as how they are mounted or integrated into the system. The fundamental goal of sensor positioning is to make sure that sensors are ideally situated to gather the necessary data to assess the system’s health information. This category further separates into structural placement, network topology and sensor location subcategories. According to the system’s structural characteristics, the first entails deciding how the sensors should be placed, the second deals with how the sensors should be set up in a network to ensure total coverage and redundancy, and the last one deals with location and interferences. Data Processing: In order to identify errors and forecast system behaviour, data processing takes into account considerations linked to how sensor data are processed, examined, and interpreted. Data processing’s primary goal is to convert sensor data into useful information that can be applied to decision-making. With good data processing, the best use of the data can be made with appropriate techniques to maximise the information gain. The three subcategories within this area are feature extraction, fault diagnosis and prognostics. To extract pertinent characteristics from sensor data, feature extraction uses time–frequency analysis, statistical techniques, and wavelet transforms. Machine learning, neural networks, and expert systems are used in fault diagnostics to find systematic flows. Prognostics include estimating the system’s remaining useful lifetime, examining degradation, and determining failure modes.

Operations: Considering how sensors are powered, how data are acquired, and how they are calibrated to preserve accuracy over time are all part of operations. The fundamental goal of operations is to make sure that sensors are accurate and performing well. There are three subcategories within this category: network lifetime, coverage efficiency, and monitoring. To ensure reliable operations, coverage efficiency over the network lifetime of the sensors should be managed precisely. It is imperative to capture the necessary information and adjust system parameters in response to changing conditions to inform decision support systems by continuous monitoring.

Overall, the proposed taxonomy provides a comprehensive framework for understanding the key concepts and considerations related to sensor optimisation in IVHM systems. This is also useful for organising and synthesising the literature, as well as for identifying patterns and trends in the research domain and guidance for future research directions. The remainder of this paper follows with a detailed review of the identified four major areas, linked with a specified cost-function proposition for each area, and ends with a conclusion including a discussion on future research directions.

## 3. Selection

Effective sensor selection can significantly improve the accuracy and reliability of health monitoring, fault diagnosis, and prognosis. For this reason, sensor selection is a challenging task that involves various considerations: the type and number of sensors, the performance characteristics of sensors, and the cost of sensor installation and maintenance.

This section presents an overview of sensor-selection methods and techniques for IVHM applications. Starting with a discussion of the selection process and frameworks available in the literature, various methods for sensor selection, such as analytical, heuristic, and machine learning-based approaches are explored. Selecting sensors based on information gain, such as mutual information-based, entropy-based, and Fisher information-based selection methods, as well as dynamic sensor-selection optimisation perspectives, is also documented.

Lastly, the evaluation of performance characteristics for sensor selection, including figures of merits, objective and cost functions, and information gain, are presented. The limitations and challenges of information-gain optimisation, such as complexity and computational requirements, lack of comprehensive models and data, and integration with other optimisation approaches and decision-making frameworks, are emphasised. Also, multi-objective optimisation and multi-criteria decision-making techniques for sensor-selection optimisation, as well as sensor redundancy and implementation techniques, are presented.

### 3.1. Sensor-Selection Methods

For clarity, it is important to note that “sensor selection” has two distinct meanings in this field. The first refers to the process of selecting sensors from an existing network to optimise their performance by determining which sensors should be active at any given time. The second meaning, which is the focus of this work, pertains to selecting sensors for integration into a system during the design and build process. In this case, the selection process is geared towards choosing the most suitable sensors for the specific task at hand [2].

Several methods have been proposed to address sensor selection for IVHM systems, ranging from heuristic and analytical approaches to machine learning-based techniques. These methods differ in terms of their underlying assumptions, computational complexity, and solution quality. The selection process involves evaluating and comparing different sensor performance characteristics, such as sensitivity, selectivity, reliability, and cost. Moreover, sensor selection should account for adaption to changing operating conditions or system states. The first sensor-selection use case was in aerospace systems, and it was based on design and performance requirements rather than a health management perspective. A model-based procedure that systematically selects an optimal sensor suite for the overall health assessment of a designated host system is described in [3]. This procedure, known as the systematic sensor-selection strategy (S4), was developed and implemented at NASA’s John H. Glenn Research Centre with the primary objective of enhancing design phase planning and preparations for in-space propulsion health management systems.

**Figure 5:**
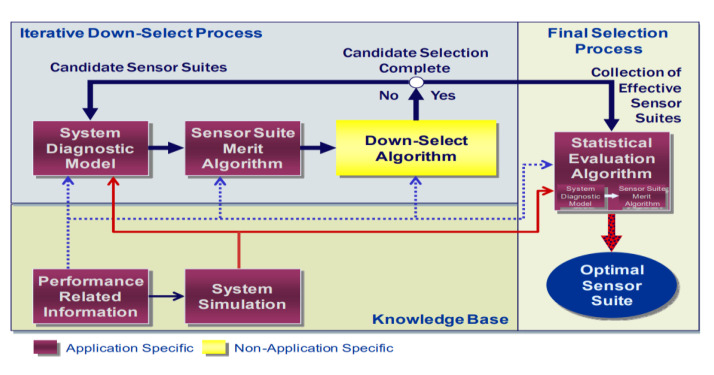
S4 Architecture [3].

**Figure 6:**
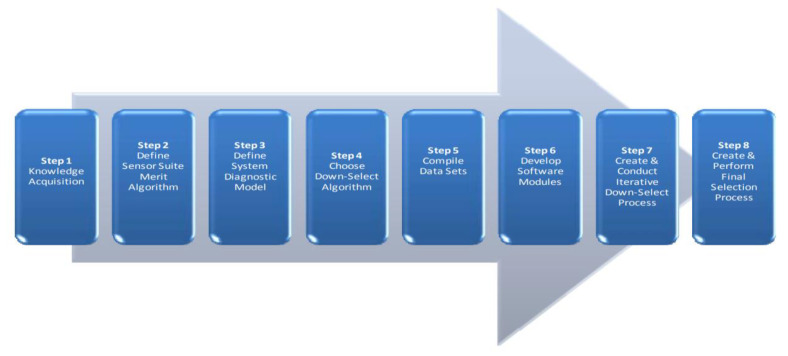
Process of applying the S4 strategy to a specific system [3].

Figure 5 illustrates the overall architecture of the S4 strategy, depicting the systematic approach to sensor selection and optimisation. On the other hand, Figure 6 provides a detailed representation of the step-by-step process for applying the S4 strategy to a specific system.

The S4 strategy encompasses a well-structured and comprehensive framework for identifying and choosing the most suitable sensors to be integrated into in-space propulsion systems. By employing a systematic methodology, it ensures that the selected sensors are aligned with the system’s requirements, performance objectives, and environmental conditions.

A general approach to sensor selection from a health management perspective was addressed in [4] with a proposed architecture that provides a justifiable, defendable sensor suite to address system health assessment requirements and additionally considers use outside of the aerospace community.

Sensor selection involves a multi-stage process that typically includes a knowledge-based selection, a down-select iteration, and a statistical evaluation algorithm. This process aims to identify the optimal set of sensors that can satisfy the requirements of the IVHM system while minimising the overall cost and complexity. The result of the analysis indicates that general sensor-selection problems addressing diagnosability, or observability, are NP-complete and are therefore computationally intractable [5]. To solve complex problems in a reasonable time, approximate search solutions are needed. Brute force or exhaustive search methods are ideal but can take too much time. Instead, refined search methods are used to find optimal or near-optimal solutions based on the objective function, which is an algorithmic representation of established figures of merit (FoMs) and system constraints.

Many techniques have been developed for general optimised solution searches. These optimisation techniques are well documented in [6], and Table 2 shows a selection of techniques found in this literature review with references.

To solve an optimisation problem, it is important to find an effective and achievable solution within a reasonable time frame. Heuristic approaches like genetic algorithms, particle swarm optimisation, and simulated annealing rely on stochastic search strategies and can yield promising results. Machine learning-based methods like decision trees, random forests, and support vector machines can also be effective in identifying the underlying patterns of a system by using a training dataset. One way to solve sensor-selection problems in the diagnostic domain is through information-gain optimisation.

Several information-gain-based methods have been proposed in the literature, such as mutual information-based methods, entropy-based methods, and Fisher information-based methods [11,16]. These methods are effective in reducing the number of sensors required while maintaining the high accuracy of health monitoring systems. While different sensor-selection methods have their own advantages and limitations, it is important to consider the underlying assumptions, computational complexity, and solution quality of each method when selecting the appropriate method for a given application.

Finally, it is worth noting that prior knowledge and uncertainty should be considered in the sensor-selection process. Prior knowledge about the system, its components, and its failure modes can provide valuable information for selecting the most informative sensors. Moreover, it is important to consider the uncertainty associated with the sensor measurements, system model, and diagnostic task when selecting sensors. Several studies have proposed methods for incorporating prior knowledge and uncertainty in the sensor-selection process, such as Bayesian approaches [17] and fuzzy logic-based methods [18].

### 3.2. Evaluation of Performance Characteristics for Selection

Figures of merit (FoMs) are quantitative measures of performance that can be used to evaluate the suitability of sensors for a particular application. FoMs provide a framework for evaluating and comparing different sensors and selecting the best sensor for a given application. FoMs can be categorised into two groups: objective FoMs and subjective FoMs.

- Objective FoMs are quantitative measures that can be directly calculated from the sensor data. They include sensitivity, specificity, accuracy, precision, and response time.
- Subjective FoMs are qualitative measures that require expert judgment. Some examples of objective FoMs include ease of use, reliability, and maintainability.

Sensor-selection methods in the diagnostic domain can be evaluated based on different performance characteristics, such as fault detection rate (FDR), fault isolation rate (FIR), false alarm probability (FAP), and correct classification rate (CCR). These performance characteristics are influenced by the choice of sensors and their arrangement. Thus, it is essential to carefully evaluate the performance characteristics of sensors before selecting them for a specific application [19].

Objective functions are mathematical functions that need to be optimised to select the best sensors. These functions are designed to maximise the performance of the system, subject to constraints such as budget, weight, and power consumption. Cost functions are also used to evaluate the cost of the sensor system and parameter trade-offs, including the cost of sensors, energy consumption, installation, maintenance, and replacement. The sensor-selection problem requires the achievement of excellent performance while minimising costs. However, these two objectives often conflict as better performance typically comes with higher costs. Thus, researchers must find the optimal balance between cost and performance.

When setting up large, complex systems, it is important to consider the cost of sensor configuration, as it often involves purchasing and installing a significant number of sensors. In addition to these upfront costs, ongoing usage costs should also be factored in, which may vary based on factors like connectivity, bandwidth, and sensor risk. Energy usage was the main objective for most of the sensor-selection studies in [19], where the focus was solely on communication energy, which consists of detecting and transmission energy. There are other variables to consider, like in [20], where the performance (fault detection reliability of aircraft engines) of the sensor was also considered as well as cost (installation and communication).

Information gain is a measure of how much a sensor measurement reduces the uncertainty about the system state. Information-gain optimisation is a method of selecting sensors that maximise the amount of information gained from sensor measurements. It has been widely used in the design of sensor networks for various applications, including health monitoring, surveillance, and environmental monitoring. When implementing information-gain optimisation in practice, several considerations need to be considered. These include cost constraints and trade-offs, sensor availability and compatibility, and robustness to noise and uncertainty. Fault trajectories, fault tolerance, fault detection, and fault isolation are essential considerations for the evaluation of information gain in diagnostics [3].

Although information-gain optimisation is a powerful tool for sensor selection, it has several limitations and challenges. One of the main challenges is the complexity and computational requirements. Information-gain optimisation involves the calculation of mutual information between sensors and system states, which can be computationally expensive for large-scale systems. Another challenge is the lack of comprehensive models and data. Information-gain optimisation requires accurate models of the system and the sensor characteristics, as well as accurate data on the system states and sensor measurements. Finally, integration with other optimisation approaches and decision-making frameworks is also a challenge. Information-gain optimisation needs to be integrated with other optimisation approaches and decision-making frameworks to ensure that the selected sensors meet the overall system requirements.

### 3.3. Multi-Objective Optimisation/Multi-Criteria Decision-Making Techniques for Sensor-Selection Optimisation

Sensor selection is often a multi-objective optimisation (MOO) problem that requires the simultaneous satisfaction of several objectives. These objectives can include diagnostic performance, robustness, and cost-effectiveness. In such cases, MOO techniques can be employed to consider the trade-offs between these objectives and to find the optimal sensor set that meets the desired criteria. In MOO, the goal is to find the Pareto-optimal solutions. A solution is Pareto-optimal if it represents the best possible trade-off between conflicting objectives. This set of Pareto-optimal solutions forms the Pareto front, which represents the boundary of the feasible solutions that cannot be further improved without sacrificing performance in other objectives. The evaluation of the Pareto-optimal solutions can help decision-makers identify trade-offs between different objectives and select the best solution based on their preferences and constraints.

Multi-criteria decision-making (MCDM) techniques such as the analytical hierarchy process (AHP) and the technique for order of preference by similarity to ideal solution (TOPSIS) can be used to rank sensors based on their diagnostic performance and other criteria. The AHP is a widely used MCDM technique that involves breaking down complex decisions into a hierarchy of objectives, criteria, and alternatives, and assigning relative weights to each level. TOPSIS is another MCDM technique that ranks alternatives by their similarity to the ideal solution and their distance from the worst solution.

Different MOO methods, such as mathematical programming, evolutionary algorithms, and hybrid methods, can be used to solve the sensor-selection problem. Mathematical programming methods, such as linear programming and mixed-integer programming, are efficient but may be limited by the complexity of the optimisation problem. Evolutionary algorithms, such as genetic algorithms [21], are robust and can handle complex optimisation problems but may require a large number of iterations. Hybrid methods [22], which combine different optimisation techniques, can provide a balance between efficiency and robustness.

**Figure 7:**
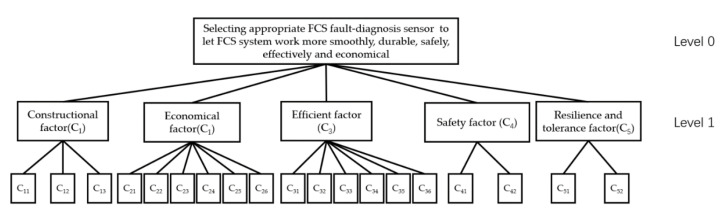
Multi-level hierarchical structure for fuel-cell-stack fault-diagnosis sensor criteria weight definition [24].

One popular method for multi-criteria decision-making is the analytical hierarchy process (AHP), which involves the decomposition of the problem into a hierarchy of objectives, criteria, and alternatives. The AHP then assigns weights to each element in the hierarchy and uses them to rank the alternatives based on their overall desirability [23]. Figure 7 show the multi-level hierarchical structure for fuel-cell-stack fault-diagnosis sensor criteria weight definition.

Another similar approach to the AHP is grey clustering, which is an essential technique for the analysis and evaluation of systems. Its efficiency in handling diverse feature types makes it indispensable in assessing sensor-suite options. The recommended course of action is to assign a grey classification to the clustering object [8].

Another commonly used technique is the TOPSIS, which involves ranking alternatives based on their similarity to an ideal solution and their distance from the worst solution. The TOPSIS has been applied to sensor selection and MOO problems in several studies [16,24,25].

The best method for sensor selection, whether it is MOO or MCDM, depends on the specific requirements and constraints of the application at hand. Each approach has its own set of advantages and limitations, and the choice should be made based on the characteristics of the problem and the goals of the sensor-selection process.

The decision on which method to choose can be influenced by the following factors:

1. Number of Objectives: If the sensor-selection problem involves multiple, conflicting objectives, MOO is well-suited to identify trade-offs and find Pareto-optimal solutions. On the other hand, if the decision primarily revolves around a single dominant criterion, MCDM may be more appropriate.
2. Complexity of the Problem: For large-scale and complex sensor-selection problems, MOO’s ability to handle multiple objectives can be advantageous. However, if the problem is relatively simple and straightforward, MCDM may provide a quicker and more straightforward solution.
3. Decision-Maker’s Expertise: If the decision-maker is familiar with MOO techniques and capable of interpreting the Pareto front, MOO could be a compelling choice. Conversely, if simplicity and transparency are essential, MCDM may be preferred.
4. Data Availability: The availability of reliable data for multiple objectives may influence the choice of method. If the data for different criteria are scarce or uncertain, MCDM might be a more practical option.

Ultimately, the best method for sensor selection will vary based on the specific context and the priorities of the decision-makers. In some cases, a combination of both MOO and MCDM might be used to leverage the strengths of each approach and arrive at a well-informed and balanced decision. Careful consideration of the problem’s complexity, objectives, and available resources is crucial in making an informed choice for the sensor-selection process.

### 3.4. Sensor Redundancy

Sensor redundancy is a crucial technique used in many industrial and engineering applications to increase the diagnostic reliability and fault tolerance of systems. By using redundant sensors to detect and isolate faults, the system’s diagnostic reliability and accuracy can be improved. Once the system is built, its reliability remains constant and cannot be changed. However, what can be managed is the system’s operational reliability or the ability to maintain its desired level of reliability over time.

This is where sensor redundancy comes into play. While we cannot change the system’s inherent reliability, we can use redundant sensors to monitor the system’s behaviour continuously and detect potential faults or deviations from the expected performance. By using redundant sensors, we can create a system of checks and balances that helps ensure the system continues to operate within acceptable performance limits.

The redundant sensors serve as a means of managing the system’s operational reliability by providing real-time data on system health and performance. If a sensor malfunctions or provides erroneous readings due to a fault, the redundant sensors can act as backups and provide accurate data. This technique involves measuring the same parameter with multiple sensors, providing a backup in case of sensor failure or malfunction. Determining the optimal number of redundant sensors is crucial for achieving the desired level of performance while minimising the cost of the system. The optimal number of redundant sensors depends on several factors, such as system criticality, the likelihood of sensor failures, and cost considerations. Reliability-based approaches, probabilistic analysis, and cost–benefit analysis are some of the techniques used to determine the optimal number of redundant sensors. Developing algorithms capable of processing data from multiple sensors and determining the correct measurement is necessary.

Implementing redundancy can be achieved through various approaches, with the voting algorithm being one of the most effective. This algorithm compares data from multiple sensors and selects the most accurate measurement, enhancing the system’s diagnostic reliability and accuracy. Recent studies have proposed innovative algorithms and methodologies to improve performance and availability of complex systems through sensor technology. One study [26] introduced a new voting algorithm that assigns priority to each sensor’s measurement in real time, allowing for accurate fault detection and isolation. Results from fault inoculation experiments demonstrated that the proposed algorithm outperforms the majority voter and enhanced weighted-average voter in terms of reliability and availability. While this algorithm is more complex and requires more comparisons and CPU time for each voting action, it can handle severe outliers and overcomes the problem of having no clear majority that exists in the majority voter. The traditional majority voting approach follows the majority decision, but it may struggle when there is no clear majority. The proposed voting algorithm, on the other hand, employs an adaptive prioritisation method to handle situations with no majority and make accurate decisions by giving higher importance to more reliable sensors in real time. This approach enhances the reliability and availability of complex systems and makes the voting algorithm more robust and effective in critical applications.

Another study [27] proposed a simple and efficient way to estimate the diagnostic coverage and false alarm values of redundant sensor systems using statistical methods. By estimating these values, this methodology enables the development of a safety concept and functional safety analysis for sensor systems in safety-critical applications. This approach can also be useful for statistical sensor system optimisations and ensuring the reliability of IVHM systems. In addition, ref. [28] proposed statistical design, estimation, and optimisation approaches for efficient product definition and the design of integrated sensor systems for safety-critical applications. This study highlights the limitations of relying on redundant configurations alone and proposes a methodology that optimises individual sensing channel performance and dependability figures, dependent on the redundant sensor output function and its diagnostic mechanism parameters. The proposed methodology was demonstrated for a redundant integrated linear-hall magnetic-field sensor system for safety-critical automotive applications, and it provides a practical method for sensor system architects to perform an overall optimisation of redundant sensor systems, including dependability requirements.

Overall, to ensure that IVHM systems are reliable and readily available, it is essential to prioritise sensor redundancy. By utilising multiple sensors, the system’s fault tolerance can be improved, and its diagnostic capabilities can be enhanced. After conducting a thorough review of the relevant literature, it is apparent that there are various strategies for optimising sensor redundancy in IVHM applications. Overall, these papers provide valuable insights into the optimisation of sensor redundancy for IVHM applications.

### 3.5. Cost Function for Selection Optimisation

This section delves into the importance of the cost function in guiding decision-making during sensor selection.

The cost function for sensor selection plays a pivotal role in achieving a balanced approach that maximises system performance while considering the associated costs. It allows engineers and decision-makers to weigh the trade-offs between sensor capabilities and their economic implications. By incorporating cost considerations into the optimisation process, a more cost-effective sensor configuration can be obtained, aligning with the project’s budgetary constraints.

A comprehensive cost function for sensor selection includes the following factors:

1. Sensor Performance: Represents the performance characteristics of the sensors, including sensitivity, selectivity, reliability, accuracy, precision, response time, and robustness. Sensor performance can be quantified based on metrics such as accuracy, precision, sensitivity, response time, or any other relevant performance criteria.
2. System Compatibility: Refers to the compatibility of the selected sensors with the target system, considering factors such as physical dimensions, communication protocols, power requirements, environmental suitability, and integration complexity. System compatibility can be quantified by assessing how well the sensors can integrate and communicate with other components of the complex engineering system. This can be measured based on compatibility protocols, communication standards, or successful integration tests.
3. Cost-Effectiveness: Addresses the cost-related considerations in sensor selection. Cost-effectiveness can be quantified by considering the total cost of ownership of the sensors, including acquisition costs, installation costs, maintenance costs, and any other associated expenses. This can be represented by a monetary value or a cost-to-benefit ratio.
4. Information Gain: Quantifies the amount of useful information that can be extracted from the sensor data for the intended application, considering factors such as data quality, relevance, comprehensiveness, and the potential for decision-making and analysis. Information gain can be quantified by evaluating how much valuable information the sensors can provide for the system. This can be measured based on metrics such as data entropy reduction, information theory, or the ability to detect and identify relevant events or patterns.
5. Sensor durability can be quantified by assessing the expected lifespan, reliability, and robustness of the sensors under normal operating conditions. This can be measured in terms of mean time between failures (MTBF) or failure rates.
6. Sensor redundancy can be quantified by assessing the level of redundancy or backup sensors in the system. This can be measured based on the number of redundant sensors available or the ability to seamlessly switch between sensors in case of failure.
7. Sensor calibration stability can be quantified by assessing the ability of the sensors to maintain consistent and accurate calibration over time. This can be measured by evaluating calibration drift or the need for frequent recalibration.
8. Sensor interoperability can be quantified by evaluating the ability of the sensors to work seamlessly with other sensors and systems within the complex engineering system. This can be measured based on interoperability protocols, data exchange capabilities, or successful integration with other components.

These approaches provide a starting point for quantifying the objective functions in the selection part of the sensor optimisation process. The first four factors are considered for the general cost function in the selection part, however, depending on the specific context and requirements of the complex engineering system, the actual quantification methods and metrics may vary.

Cost (f) = α × Sensor Performance + β × System Compatibility + γ × Cost Effectiveness + δ × Information Gain

To quantitatively represent the cost function (cost (f)), appropriate weights are assigned to each cost component based on their relative importance within the complex engineering system. These weights can be determined through rigorous cost–benefit analyses, considering factors such as the project budget, expected sensor lifespan, and specific operational requirements. By employing multi-objective optimisation techniques, the cost function can be effectively integrated with other objective functions, such as sensor performance and compatibility, to obtain an optimal and cost-effective sensor configuration.

By incorporating a well-defined cost function into the sensor-selection process, complex engineering systems can make informed decisions that strike the right balance between sensor capabilities and economic considerations. The cost-optimised sensor configuration contributes significantly to the overall success and sustainability of the system.

## 4. Placement

Optimal sensor placement (OSP) aims to determine the optimal number and location of sensors to be deployed in a system while considering various factors such as cost, measurement accuracy, and system performance. The use of OSP is particularly important in systems where the placement of sensors can significantly impact diagnostic accuracy and the performance of the system it is monitoring. For instance, in fault diagnosis systems, improper sensor placement can lead to the inaccurate detection and diagnosis of faults.

### 4.1. Theoretical Background and OSP Methods

Various theoretical principles are used in OSP, including statistical analysis, mathematical modelling, and simulation techniques. One key aspect of OSP is understanding the key parameters that affect optimal sensor placement and the trade-offs between objectives and constraints. Another important aspect is the use of inverse problems to determine the location of the source of a signal from sensor measurements. Modelling sensor data using the Gaussian process (GP) is also a commonly used technique in OSP, which is a natural generalisation of linear regression that allows for the consideration of uncertainty about predictions. A comparison of the main used techniques in OSP is shown in Table 3.

Nakai et al. [7] discussed different approaches to sensor-placement optimisation for various applications. The focus was on selecting the optimal set of sensors to estimate high-dimensional data based on different optimality criteria, such as D-, A-, and E-optimality, which are used to maximise the determinant, minimise the trace of the inverse, and maximise the minimum eigenvalue of the Fisher information matrix, respectively. The performance of the greedy algorithms based on these criteria was evaluated using randomly generated systems and a practical dataset related to climate science. A comparison of the pros and cons of the two approaches is stated in Table 4.

Wan et al. [29] reviewed the optimal sensor placement for aircraft structural health management (ASHM), which mainly focuses on structural health monitoring and assessment, microstructure fault monitoring and isolation, overload, corrosion monitoring, and residual life assessment. The authors emphasise the importance of OSP for ASHM and discuss the difficulty of optimising sensor placement in large aircraft structures. The study proposes the use of singular value decomposition (SVD), QR decomposition, and fuzzy measurement coverage to optimise sensor measuring points. The final result of the OSP is verified through QR decomposition and fuzzy measurement coverage, and the scheme of OSP is analysed for aircraft wing structure. A comparison of the approaches is shown in the Table 5.

Krause et al. [30] also discussed the problem of choosing sensor locations when monitoring spatial phenomena modelled as Gaussian processes (GPs). They note several common strategies and tackle the combinatorial optimisation problem of maximising the mutual information between the chosen locations and the locations that are not selected. The paper proves that this problem is NP-complete but describes a polynomial-time approximation that is within (1 − 1/e) of the optimum by exploiting the sub-modularity of mutual information. The paper extends its algorithm to exploit lazy evaluations and local structure in the GP, yielding significant speedups. It also extends the approach to find placements that are robust against node failures and uncertainties in the model, again exploiting the sub-modularity of the objective function. Finally, the paper demonstrates the advantages of the approach towards optimising mutual information in an extensive empirical study on two real-world data sets. A comparison of the approaches shown in Table 6.

The reviewed studies emphasise the importance of OSP in improving fault diagnosis efficiency, sensor layout selection, and ASHM. Each study proposes a different approach to optimising sensor placement, including the use of dynamic fault tree, dynamic Bayesian network, SVD, QR decomposition, and fuzzy measurement coverage. They demonstrate the complex nature of OSP and highlight the need for a systematic approach to sensor placement, considering different criteria and factors such as the number of sensors, edge effect, measurement degree of freedom, similarity of sensor locations, and hiddenness of fault position, and provide insights that can be applied to various fields such as aerospace, energy, and transportation.

OSP methods have been widely used in various fields to solve the sensor placement problem. These methods can be broadly categorised into three groups: heuristic, evolutionary and deterministic. The optimisation of sensor placement is a challenging task that involves finding the optimal locations for sensors based on a set of objectives and constraints. A range of optimisation techniques has been developed to address this problem, including sensitivity-based and topology-based approaches, as well as linear and non-linear optimisation methods.

Some of the most commonly used optimisation techniques for sensor placement include evolutionary algorithms, particle swarm optimisation, and greedy algorithms. Evolutionary algorithms, such as genetic algorithms or differential evolution, are based on the principles of natural selection and survival of the fittest. These algorithms generate a population of candidate solutions and iteratively improve them by applying genetic operators, such as mutation, crossover, and selection. Particle swarm optimisation is a population-based optimisation technique inspired by the behaviour of bird flocks or fish schools. It involves iteratively adjusting the position and velocity of a set of particles to find the optimal solution. Greedy algorithms are simple heuristic techniques that aim to find the optimal solution by iteratively adding or removing sensors based on a set of criteria, such as the information gain or the cost–benefit ratio.

Sensitivity-based approaches aim to identify the most sensitive locations in a system by analysing the response of the system to changes in the input parameters. This involves calculating the sensitivity coefficients, which describe how changes in the sensor measurements affect the system output. These coefficients are used to guide the placement of sensors to maximise the system’s response to changes in the input parameters.

Topology-based approaches focus on identifying the most critical locations in a system by analysing the topology of the system. This involves identifying the most important nodes, edges, or regions in the system using graph-based techniques, such as centrality analysis or clustering. These techniques can be used to guide the placement of sensors to maximise the coverage of the critical regions in the system.

In [31], Clark et al. discussed the problem of optimal sensor placement under a cost constraint, which arises in various industrial and scientific applications. A well-established greedy algorithm for optimal sensor placement without cost constraints is extended to incorporate cost constraints, and the algorithm’s effectiveness was demonstrated on datasets related to facial recognition, climate science, and fluid mechanics. The paper emphasises that the cost-error landscape varies by application, and intuitive connections to underlying physics are observed.

Gomes et al. [32] examined the problem of identifying structural damages in large-scale structures, mainly in aerospace applications. A metaheuristic algorithm called the “firefly algorithm” (FA) is used to identify structural damages by solving an inverse problem. The Fisher information matrix is used to optimise sensor placement, and the results demonstrate that optimised sensors contribute to the improved identification of damages, especially in complex and large-scale structures. The proposed optimised damage identification process using FIM-FA has the potential to be extended to a wide range of structural health monitoring (SHM) applications in complex structures, where traditional non-destructive inspection methods may not be practical due to the complexity and restricted access to the structure.

Feng et al. [24] reviewed the development of an OSP scheme to improve fault diagnosis efficiency considering common cause failure. The study introduces a dynamic fault tree converted to a dynamic Bayesian network to calculate reliability parameters and construct the decision matrix. An efficient TOPSIS algorithm is adopted to determine the potential sensor locations. A diagnostic sensor model is also developed to take into account the failure sequence between a sensor and a component. The authors provide a case study to prove the significant impact of common cause failure on sensor placement.

Yang et al. [33] focused on the investigation of OSP for a multi-rotary-joint solar power satellite (MJ-SPS) using six OSP methods to select the best sensor layout. Three standards and two novel criteria, i.e., sensor distribution and similarity of sensor locations, are added to evaluate the effectiveness of the sensor configurations. The study emphasises the importance of the work for the MJ-SPS and OSP methods, comparing different numbers of sensors and orders of modal shape.

It is important to note that the optimal sensor placement depends not only on the system’s objectives but also on the type and number of sensors available, as well as the impact of environmental factors on sensor performance. For instance, the placement of temperature sensors in a heat exchanger may differ depending on the type of temperature sensor used (e.g., thermocouple, RTD, or infrared sensor) and the impact of fouling or corrosion on sensor accuracy.

Overall, the choice of sensor-placement optimisation method depends on the complexity of the problem, the computational resources available, and the required level of solution accuracy. Each method has its advantages and disadvantages, and the optimal method should be selected based on the specific requirements of the problem. A comparison of the methods is shown in Table 7.

### 4.2. Sparsity and Data-Driven Learning

In this section, we discuss the use of sparsity and data-driven learning techniques in OSP. Sparsity has been extensively used to improve the performance of inverse problems by reducing the number of unknowns and increasing the robustness of the solution. Sparsity is a well-known concept in data science and optimisation theory. This concept has been widely applied in various fields, including image and signal processing, machine learning, and optimisation [34,35,36].

In sensor-placement optimisation, sparsity can be used to identify important measurement points in complex systems, leading to significant cost savings in the number of sensors required. Data-driven learning approaches, such as compressed sensing and dimensionality reduction, have been used to achieve sparsity in sensor placement [37]. These methods have been successfully applied to a variety of applications, including structural health monitoring, power-systems health monitoring, and water distribution networks.

Compressed sensing is a mathematical technique that allows for the recovery of a sparse signal from a small number of measurements. Compressed sensing has also been used in conjunction with other optimisation methods, such as convex optimisation, to improve sensor placement performance. Dimensionality reduction is another data-driven approach that can be used for sensor-placement optimisation. Dimensionality reduction is the process of reducing the number of variables in a dataset while retaining the most important information. This can be achieved using techniques such as principal component analysis (PCA), proper orthogonal decomposition (POD) or T-distributed stochastic neighbour embedding (t-SNE). It can be used to identify important measurement points in a system and reduce the number of sensors required.

When dealing with nonlinear systems, standard techniques for feature selection and sensor placement that rely on linearity assumptions or simple statistical models can result in costly oversensing without guaranteeing the recovery of desired information from the measurements. To this end, Otto et al. [38] discuss the importance of sensor placement and feature selection in solving inverse problems in nonlinear systems and highlight the limitations of existing techniques that rely on linearity or simple statistical models. To overcome these limitations, the authors propose a novel data-driven approach based on secant vectors between data points for a general type of nonlinear inverse problem. The approach is used to develop three efficient greedy algorithms that provide different robust and near-minimal reconstruction guarantees. The algorithms are demonstrated on two problems where linear techniques fail: sensor placement for reconstructing a fluid flow with a complex shock-mixing layer interaction and selecting fundamental manifold learning coordinates on a torus.

Overall, the use of sparsity and data-driven learning techniques in OSP offers new opportunities for improving the accuracy and efficiency of solutions, especially in applications involving large datasets and incomplete measurements.

### 4.3. Case Studies in Placement Optimisation

Sensor-placement optimisation has been widely applied in various fields, including structural health monitoring (SHM), power-systems health monitoring, water distribution networks, non-destructive evaluation (NDE), condition-based maintenance (CBM), and prognostics and health management (PHM). In this section, an overview of some case studies that have implemented sensor-placement optimisation techniques is given.

Structural health monitoring (SHM) is a field that aims to provide real-time information on the health condition of structures to ensure their safety and prevent catastrophic failures. The use of sensor-placement optimisation in SHM has been widely investigated in the literature. For example, Ostachowicz et al. [39] presented an unbiased state-of-the-art review of the research carried out in this area for researchers and practitioners in the SHM and optimisation fields. The review covers the definition of the optimisation problem, classification of techniques used, optimisation algorithms applied, and multi-objective optimisation. The authors of the reviewed article have focused on three commonly accepted and widely used techniques in the SHM community, which are vibration-based monitoring, strain monitoring, and elastic wave-based monitoring.

Power systems are critical infrastructures that require constant monitoring to ensure their reliability and prevent blackouts. Sensor-placement optimisation has been applied in power-systems health monitoring to improve the accuracy and efficiency of fault detection and diagnosis. A Bayesian belief network (BBN)-based approach has been proposed to optimise sensor placement for power-systems health monitoring in the work by Pourali et al. [40]. The approach utilises functional topology, physical models of sensor information, and Bayesian inference techniques, along with constraints, to determine optimised sensor placement based on information metric functions. The methodology aims to address important questions such as inferring the health of a system or subsystem with limited monitoring points, using upward, downward, or distributed propagation techniques. The dynamic BBN serves as the engine for projecting the health of the system. Such approaches are critical for ensuring effective power-systems health monitoring while minimising costs associated with excessive sensor placement.

Water distribution networks are critical infrastructures that require constant monitoring to ensure their safety and prevent leaks and contamination. Sensor-placement optimisation has been applied in water distribution networks to improve the efficiency and accuracy of leak detection and localisation. For example, Aral et al. [41] proposed a simulation-optimisation approach based on a single-objective function. The proposed model incorporates multiple factors used in the design of the system to mimic a multi-objective approach and provides the final design without specifying a preference among the multiple objectives. A reliability constraint concept has also been introduced in the optimisation model to identify the minimum number of sensors and their optimal placement required to meet a pre-specified reliability criterion for the network. A progressive genetic algorithm approach has been utilised for the solution of the model by evolving subdomain sets of the complete set of junctions present in the system. The algorithm has been tested in two networks and compared with the outcome of other solutions presented in a water distribution systems analysis symposium, showing promising results for effective water sensor-placement optimisation.

In a recent study, Kim et al. [42] discuss utilising the convolution neural network (CNN) algorithm for the NDE of aluminium panels. The objective is to classify the locations of defects by exciting the panel to generate ultrasonic Lamb waves, capturing the data through a sensor array, and then utilising deep learning to identify the features of 2D reflected waves from the defects. The study also explores the impact of optimal excitation location and sensor placement to improve the performance of the method. To ensure the training model’s robustness and effective feature extraction, experimental data are collected by slightly varying the excitation frequency and defect location. The algorithm delivers high accuracy in classifying each defect location, even when a bar is attached to the panel.

PHM plays a crucial role in ensuring the safety and reliability of aerospace systems. Design for testability (DFT) is an important consideration for improving PHM performance, as information sensing and testing are the foundation of PHM. However, traditional DFT approaches, which only focus on fault detection and isolation requirements, are inadequate for sensor design and optimisation for PHM. To address this issue, a process for sensor selection and optimisation for PHM is proposed by Yang et al. [43]. A qualitative analysis of the intrinsic requirements of PHM for testability and a quantitative definition of corresponding testability indexes are presented. Fault detection uncertainty is systematically analysed from various perspectives, including fault attributes, sensor attributes, and fault-sensor matching attributes. Object and constraint models for the sensor optimisation selection problem are studied in detail, and a sensor optimisation selection model is developed for aerospace system health management. The model considers sensor total cost as the objective function and the proposed testability indexes under uncertainty test as constraint conditions. As the model is NP-hard, a generic algorithm (GA) is introduced to obtain the optimal solution.

### 4.4. Cost Function for Placement Optimisation

This section focuses on the significance of the cost function in guiding decision-making during sensor placement.

The cost function for sensor placement serves as a valuable tool in achieving an optimal sensor deployment that maximises system performance while considering the associated costs. By incorporating cost considerations into the placement optimisation process, engineers and decision-makers can make well-informed choices that align with budgetary constraints, ensuring cost-effectiveness without compromising system functionality.

A comprehensive cost function for sensor placement includes the following factors:

1. Sensor Coverage: Quantifying the extent to which sensors capture relevant information within the system’s operational area. This includes assessing the spatial coverage and the quality of information gathered by the sensors. Sensor coverage can be quantified by assessing the spatial or temporal area covered by the sensors. This can be measured using metrics such as percentage coverage, spatial resolution, or time interval between data collection.
2. Sensor Connectivity: Evaluating the strength and reliability of sensor connections within the system. This encompasses metrics such as signal strength, connection success rate, and communication robustness. Sensor connectivity can be quantified by evaluating the ability of the sensors to establish and maintain reliable communication within the system. This can be measured based on metrics such as connection success rate, latency, or signal strength.
3. Interference Minimisation: Assessing the ability of the sensor placement to mitigate interference sources and maintain signal integrity. This includes considering interference rejection ratio, signal-to-interference ratio, and the effectiveness of interference mitigation techniques. Interference minimisation can be quantified by assessing the ability of the sensors to reduce or mitigate the impact of interference sources. This can be measured based on metrics such as signal-to-interference ratio, interference rejection ratio, or the ability to operate in noisy environments.
4. Resource Utilisation: Accounts for the efficient usage of system resources by the sensors, such as power consumption, bandwidth utilisation, and computational requirements. Resource utilisation can be quantified by evaluating how efficiently the sensors utilise system resources such as power, bandwidth, or processing capacity. This can be measured based on resource consumption rates or resource allocation efficiency.
5. Scalability can be quantified by assessing the ability of the sensor placement to accommodate system expansion or changes in the system’s scale. This can be measured based on the ease of adding or removing sensors, as well as the impact on overall system performance.

These approaches provide a starting point for quantifying the objective functions in the placement part of the sensor optimisation process. The first four factors are considered for the general cost function in the placement part; however, depending on the specific context and requirements of the complex engineering system, the actual quantification methods and metrics may vary.

Cost (f) = α × Sensor Coverage + β × Sensor Connectivity + γ × Interference Minimisation + δ × Resource Utilisation

Quantifying the cost function for sensor placement involves assigning appropriate weights to each cost component based on their relative importance within the specific complex engineering system. These weights are determined through a thorough analysis, considering factors such as project budget, resource limitations, and the system’s operational requirements. By employing multi-objective optimisation techniques, the cost function can be effectively integrated with other objective functions, such as sensor coverage, connectivity, and interference minimisation, to obtain an optimal sensor placement configuration.

By incorporating a well-defined cost function into the sensor-placement optimisation process, complex engineering systems can achieve an optimal sensor deployment that maximises system performance while adhering to budget constraints. The cost-optimised sensor placement contributes significantly to the overall efficiency and success of the system.

## 5. Data Processing

Effective data processing is a critical component in the optimisation of sensor systems, aiming to maximise the information obtained. By employing advanced techniques, data processing enables the extraction of meaningful insights, leading to improved system performance and decision-making. This section explores various approaches and methodologies used in data processing to enhance the information gained from sensor data. The full potential of sensor data can be achieved by optimising the information gained from sensor data through effective signal processing, feature extraction and selection, machine learning techniques, and data fusion. In the following sections, we delve into each of these approaches to understand their contributions in maximising the useful information obtained from sensor data.

### 5.1. Signal Processing Techniques

Signal processing techniques are employed to enhance the quality of sensor signals by reducing noise, amplifying relevant features, and mitigating interference. Through these techniques, engineers can maximise the information gained from sensor data, leading to improved system performance and more accurate analysis. The following techniques are discussed in this section: filtering techniques, time–frequency analysis techniques and waveform feature extraction techniques.

One of the most used noise reduction techniques, filtering, which includes low-pass, high-pass, and band-pass filters, is applied to remove unwanted noise and artefacts from the sensor signals. By selectively attenuating or amplifying specific frequency components, filtering improves the signal-to-noise ratio and enhances the quality of the acquired data. This allows for a more precise analysis and interpretation of the sensor measurements. The Butterworth filter is a commonly used low-pass filter that removes high-frequency noise. Another widely utilised technique is the Kalman filter, which recursively estimates the system state based on sequential measurements, effectively filtering out noise. Additionally, adaptive filters are employed to adjust their parameters in response to changing noise conditions, allowing for the tracking of time-varying signals. In an optimal filtering example of a stochastic singular system with correlated noises presented by Sun et al. [44], all results generalise the Kalman filter and its effectiveness, presented in a simulation example.

Time–frequency representation (TFR) has been a field of active research in the last few decades and continues to be a subject of interest today. A precise and accurate representation of nonstationary signals in the time–frequency domain is crucial, particularly in mechanical fault diagnosis. Traditional TFRs depict the energy or power of signals in two-dimensional functions of time and frequency, effectively capturing fault signatures in diagnostic applications. Various TFR methods employ different kernel functions, such as the short-time Fourier transform (STFT) with a linear kernel, the Wigner–Ville distribution (WVD) with a quadratic kernel, and the wavelet transform, which utilises an analysis basis constrained in both time and frequency [34].

Waveform feature extraction techniques are employed to extract relevant features from sensor data. Peak detection is a commonly used technique that identifies the maximum or minimum values within a waveform, providing insights into signal characteristics [45]. Zero-crossing detection identifies the points at which a waveform crosses the horizontal axis, offering information about signal behaviour. Fourier analysis, which decomposes a signal into its frequency components using the Fourier transform, enables further analysis and interpretation for larger datasets [46].

In summary, signal processing techniques are essential for optimising the information gained from sensor data. By effectively applying these signal processing techniques, engineers can extract valuable insights, improve system performance, and make informed decisions based on the processed sensor data.

### 5.2. Feature Extraction and Selection

Feature extraction and selection methods are utilised to identify and extract relevant information from the processed sensor data. By focusing on key features, these techniques reduce the dimensionality of the data and the computational burden associated with processing large datasets, enhance computational efficiency, and highlight the most informative aspects.

Feature extraction is an important step in the data processing phase of the basic condition monitoring process. This extraction process is particularly important for handling noisy sensor data and avoiding excessive input features, especially in the case of vibration data, during the classifier learning phase. Therefore, feature extraction is often considered the first and essential step in any classification task [47].

Common basic features include maximum, mean, minimum, peak, peak-to-peak interval, and others. Additionally, more complex feature extraction methods like principal component analysis (PCA), independent component analysis (ICA), and kernel principal component analysis (KPCA) can be employed [48]. These advanced methods enable the extraction of more intricate and informative features from the sensor data, enhancing the accuracy and effectiveness of classification algorithms.

Feature selection has gained significant attention in recent years in machine learning applications. Its objective is to identify and retain the most relevant features from an original dataset, aiming to enhance the quality and efficiency of feature sets used in various tasks such as classification, regression, clustering, and time-series prediction. This objective can be achieved using a variety of methods, including filter, wrapper, and embedded approaches.

**Figure 8:**
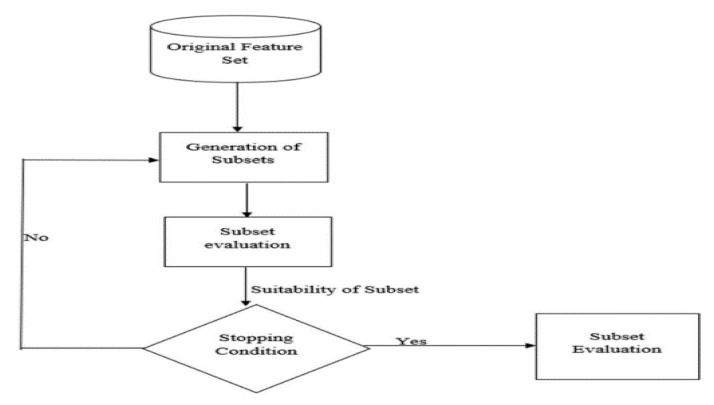
Illustration of the general feature selection process [50].

Irrelevant or redundant features can lead to overfitting and performance degradation, making feature selection essential for mitigating these issues. By reducing the dimensionality of the dataset and selecting the most informative subset of features, feature selection techniques offer benefits such as improved interpretability of models, reduced computational costs, and enhanced learning accuracy. These techniques have found widespread adoption across domains like text mining, image analysis, and biomedical research. The visual representation of the feature selection process, as depicted in Figure 8, showcasing the transformation of an original feature set into a carefully selected subset, resulting in improved performance and efficiency of machine learning algorithms [49].

Filter approaches involve ranking features based on statistical measures such as correlation or mutual information. Wrapper approaches evaluate the performance of a specific machine learning algorithm using different subsets of features and select the subset that yields the best performance. Embedded approaches involve selecting features as part of the training process for the machine learning algorithm [50].

Several feature extraction and selection techniques have been applied in complex systems, each with its own strengths and limitations. In a study on feature selection for pattern classification systems, Peng et al. [51] investigated the use of the minimal-redundancy-maximal-relevance criterion (mRMR) based on mutual information. Their goal was to select a compact set of superior features at a low cost. The proposed approach involved a two-stage feature selection algorithm that combined mRMR with other advanced feature selectors, such as wrappers. The algorithm was extensively evaluated using different classifiers (naive Bayes, support vector machine, and linear discriminate analysis) and diverse datasets (handwritten digits, arrhythmia, NCI cancer cell lines, and lymphoma tissues). The experimental results demonstrated the promising improvement in feature selection and classification accuracy achieved by incorporating mRMR.

The choice of feature extraction and selection technique depends on various factors, including the specific problem being addressed, the type of data being analysed, and the available computational resources. It is crucial to evaluate the performance of different techniques in the context of the given application to determine the most suitable technique.

### 5.3. Machine Learning Techniques

Machine learning (ML) techniques have emerged as powerful tools for data processing in sensor systems. By leveraging algorithms such as classification, regression, and anomaly detection, machine learning can automatically learn patterns and relationships within sensor data. This enables the system to make predictions, detect anomalies, and uncover complex insights that might not be immediately apparent. Integrating machine learning into data processing enhances the system’s ability to extract valuable information and optimise the sensor system’s performance.

In the field of PHM, data-driven methods, particularly ML and deep learning (DL) techniques, have gained widespread adoption for tasks such as anomaly detection, fault diagnostics, and prognostics [52]. These methods possess the capability to handle large volumes of highly nonlinear data effectively. DL models excel at processing operational data and automatically generating features for various tasks, including detection, classification, and prediction of patterns within the data. This reduces the reliance on domain expertise and extensive manual feature engineering, particularly when complete and representative data are available.

Learning problems in the context of DL can be categorised into four main groups: supervised, unsupervised, semi-supervised, and reinforcement learning. To implement DL algorithms, three key components are required: (1) training and testing data, (2) an objective function, and (3) an optimisation scheme. Variations in these components give rise to a multitude of distinct DL algorithms and architectures, such as convolutional neural networks (CNNs), recurrent neural networks (RNNs), long short-term memory networks (LSTMs), generative adversarial networks (GANs), and autoencoders [52].

Supervised learning involves learning from labelled datasets to make predictions or classifications based on observed patterns. Unsupervised learning, on the other hand, focuses on finding hidden patterns and structures within unlabelled data. Semi-supervised learning combines labelled and unlabelled data to leverage both sources of information. Finally, reinforcement learning involves learning through interactions with an environment, where the algorithm receives feedback in the form of rewards or punishments to optimise its actions. Each type of learning offers a range of capabilities and is applicable to various tasks within complex engineering systems. The selection of the most suitable technique depends on the nature of the problem, the available data, and the desired outcomes.

Rezaeianjouybari et al. [53] presented the state-of-the-art, challenges, and opportunities for deep learning applications in PHM. Well-documented lists of activation functions in deep learning, optimisation algorithms in deep architectures, regularisations in training deep networks, performance metrics for PHM model evaluation, public datasets for system health management, and mainstream deep learning tools were presented. The work conducted is commendable and valuable for the PHM community. The comprehensive survey on deep learning applications in PHM provides a solid foundation for researchers and practitioners interested in leveraging advanced machine learning techniques for system health monitoring and prognostics. The insights into performance metrics tailored for PHM model evaluation enable researchers to assess the effectiveness of their models accurately.

In the context of data-driven predictive maintenance (PdM), the use of machine learning (ML) algorithms has gained attention for its potential in artificial intelligence. However, the performance of ML algorithms can be compromised when dealing with high-dimensional and discontinuous machine data. Standard dimension reduction techniques may not effectively handle such challenges. To address this, Aremu et al. [54] proposed an ML-based dimension reduction framework that clusters observations based on data modality and utilises Laplacian eigenmaps embedding to obtain low-dimensional representations. The framework is applied to the Commercial Modular Aero-Propulsion System Simulation dataset, demonstrating its effectiveness in handling high-dimensional discontinuous machine data for ML-based PdM analysis.

Thoppil et al. [55] discussed popular deep learning architectures and their significance in machinery health prognostics, using benchmark time-series machinery failure datasets and highlighting the contributions of researchers in implementing deep learning approaches for accurate machinery health diagnostics and prognostics. However, the need for large-scale machinery failure data, the challenges of hyper-parameter optimisation and architecture design, and the black-box nature of deep learning algorithms remain limitations to be addressed.

Saufi et al. [56] discussed deep learning models and their applications in machinery fault detection and presented classification tables for the models used in different diagnosis stages, such as fault detection, fault identification, fault size estimation and fault growth prediction.

Intelligent fault diagnosis (IFD) has gained attention for automating machine fault recognition and reducing human labour. However, existing reviews lack comprehensive coverage and future guidelines. To address this, Lei et al. [57] provided a systematic review and roadmap of IFD’s development, encompassing traditional machine learning theories, the advent of deep learning, and the prospects of transfer learning. The roadmap highlights potential research trends and challenges in IFD. Yang et al. [58] also presented a detailed textbook about the foundation of transfer learning and its applications.

Overall, the choice of ML technique depends on the specific application and the characteristics of the data. It is important to carefully evaluate different techniques and select the most appropriate one for the problem at hand.

### 5.4. Data Fusion Techniques

**Figure 9:**
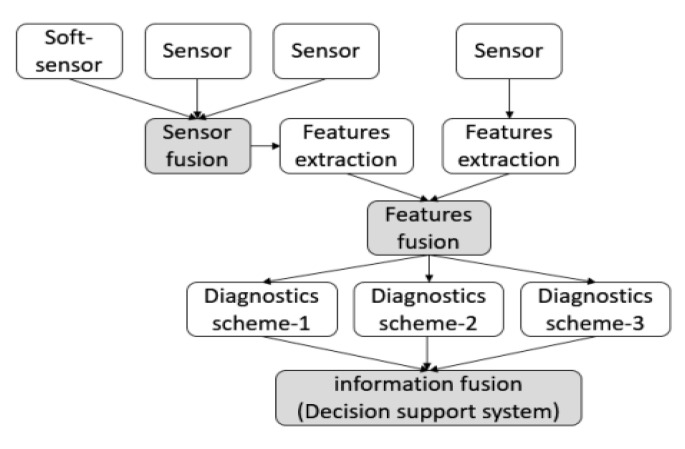
Three levels of information fusion for diagnostic and decision support systems.

Data fusion techniques are widely used in complex systems to improve the accuracy, reliability, and robustness of the results by integrating information from multiple sources. Data fusion can be performed at different levels, such as sensor-level fusion, feature-level fusion, and decision-level fusion, depending on the application requirements and the available data. Figure 9 represents three levels of information fusion techniques used in diagnostic and decision support systems. The selection of the appropriate fusion technique depends on the characteristics of the data, the desired outcome, and the computational resources available.

In the realm of sensor data management, the efficient handling of data from multiple sources is paramount. The integration and management of multi-source sensor data pose unique challenges and opportunities for optimising data quality and information extraction. When dealing with diverse sensor types and sources, the harmonisation of data formats, synchronisation, and alignment become crucial tasks. Additionally, strategies for addressing data redundancy and ensuring data integrity play a pivotal role. The selection of appropriate fusion techniques, such as sensor fusion algorithms and data integration frameworks, becomes instrumental in achieving comprehensive and meaningful insights from heterogeneous sensor data sources. By effectively managing multi-source sensor data, organisations can unlock the full potential of their sensor networks, enhancing the robustness and reliability of their data-driven decision-making processes. In this section, we delve into the intricacies of multi-source sensor data management.

Sensor-level fusion combines the raw data from multiple sensors (multi-source sensors) to form a more accurate and reliable representation of the underlying physical phenomenon. It is particularly useful in situations where the sensors have different response characteristics or are affected by different sources of noise. Sensor-level fusion can be achieved using techniques ranging from simple weighted averages to more advanced methods such as fuzzy logic, Kalman filters, and probabilistic approaches. Several studies have demonstrated the effectiveness of sensor data fusion in various applications [59].

Feature-level fusion combines the extracted features from multiple sensors to form a more informative and discriminative representation of the underlying physical phenomenon. Feature-level fusion can be achieved by concatenating, weighting, or selecting the most informative features based on their relevance and redundancy. Feature-level fusion is particularly useful in situations where the sensors have different measurement modalities or provide complementary information. In the context of vibration monitoring, feature-level fusion has been successfully applied for sensor fault detection, combining correlated measurements through statistical feature extraction [60].

At the decision level of the diagnostic and prognostic process, the fusion of results from multiple independent methods can enhance the accuracy and confidence of estimations. Different techniques may be more effective in identifying specific problems, making their combination valuable. Decision-level fusion can be achieved by voting, weighting, or selecting the most reliable algorithm or model based on their performance and the uncertainty of the results. It is particularly useful in situations where the algorithms or models have different assumptions or are trained on different data. Additionally, estimations from these methods can be combined with other sources of information such as vibration analysis, maintenance history, observations during inspections, and negative information. These fusion approaches occur at two levels: the automated decision level and the supervised decision level. While significant research has been conducted on the automated decision level, there is a scarcity of examples in the literature regarding the supervised decision level [61].

Overall, in a self-organised distributed system, collective decision-making plays a crucial role. Various approaches have been proposed for collective decision-making, including voting models, swarm methods inspired by biology, and methods for task and role allocation. However, decentralised information fusion systems face a significant challenge in terms of real-time communication. Current real protocols rely on a central control unit to manage communication timing and flow. Overcoming this challenge is essential for the development of future approaches to decentralised information fusion systems [62].

### 5.5. Cost Function for Data Processing Optimisation

This section highlights the significance of the cost function in guiding decision-making during data processing.

A comprehensive cost function for data processing includes the following factors:

1. Data Accuracy: Quantifying the level of agreement between sensor measurements and ground truth values, ensuring that the processed data are reliable for decision-making. Data accuracy can be quantified by assessing the level of agreement between sensor measurements and ground truth values or reference data. This can be measured using metrics such as mean absolute error, root-mean-square error, or statistical measures of accuracy.
2. Computational Efficiency: Evaluating the efficiency of data processing algorithms and techniques to minimise resource consumption, such as processing time, memory usage, and energy consumption. Computational efficiency can be quantified by evaluating the computational resources required for processing sensor data. This can be measured based on metrics such as processing time, memory usage, or energy consumption during data processing.
3. Feature Relevance: Assessing the significance of extracted features from sensor data to ensure that the most relevant and informative features are utilised for analysis. Feature relevance can be quantified by assessing the significance of extracted features from sensor data for the intended analysis or decision-making process. This can be measured based on metrics such as feature importance scores, information gain, or correlation coefficients.
4. Model Complexity: Considering the complexity of data processing models to strike a balance between accuracy and computational overhead, avoiding overly complex models that might be resource-intensive. Model complexity can be quantified by evaluating the complexity or simplicity of the models used for data processing. This can be measured based on metrics such as the number of parameters, the depth of the model, or the computational complexity of the algorithms.
5. Sensor Data Integration can be quantified by assessing the ability to combine and merge data from multiple sensors to create a comprehensive view of the system. This can be measured based on the effectiveness of data fusion algorithms, data alignment accuracy, or the quality of integrated data outputs.
6. Sensor Data Privacy can be quantified by evaluating the level of protection and confidentiality applied to sensor data. This can be measured based on privacy-preserving techniques, encryption methods, or compliance with data privacy regulations.

These approaches provide a starting point for quantifying the objective functions in the data processing part of the sensor optimisation process. The first four factors are considered for the general cost function in the data processing part; however, depending on the specific context and requirements of the complex engineering system, the actual quantification methods and metrics may vary.

Cost (f) = α × Data Accuracy + β × Computational Efficiency + γ × Feature Relevance + δ × Model Complexity

Quantifying the cost function for data processing involves assigning appropriate weights to each cost component based on their relative importance within the specific complex engineering system. These weights are determined through comprehensive evaluations, considering factors such as data processing performance requirements, available computational resources, and budget constraints. By employing multi-objective optimisation techniques, the cost function can be effectively integrated with other objective functions, such as data accuracy, computational efficiency, feature relevance, and model complexity, to achieve an optimal data processing configuration.

By incorporating a well-defined cost function into the data processing optimisation process, complex engineering systems can achieve an efficient and cost-effective utilisation of sensor data. The cost-optimised data processing contributes significantly to the overall efficiency and decision-making capabilities of the system.

## 6. Operation

The operation of sensor optimisation is a multifaceted process with specific objectives aimed at enhancing the overall performance and efficiency of sensor systems within complex applications. The operation of sensor optimisation is a multifaceted process with specific objectives aimed at enhancing the overall performance and efficiency of sensor systems within complex applications. The primary purpose of the operation side of sensor optimisation is to strategically select, configure, and calibrate the sensor in service to maximise its utility. This process involves several key objectives, such as data quality improvement, resource optimisation, robustness and fault tolerance, adaptability to changing conditions, and data fusion and integration.

The fundamental goal of operation is to make sure that sensors are accurate and performing well. This can include sub-categories such as monitoring and control, maintenance optimisation, fault diagnosis and prognosis, and performance optimisation. It is imperative to capture the necessary information and adjust system parameters in response to changing conditions to inform decision support systems by continuous monitoring. In summary, the operation of sensor optimisation serves the overarching purpose of improving the effectiveness and efficiency of sensor systems within complex applications.

### 6.1. Monitoring and Control

Real-time monitoring and control are critical aspects of complex systems. The use of sensors and data analytics enables the real-time monitoring and control of systems, providing continuous feedback on the system’s health and performance. By monitoring system parameters in real time, faults can be detected and addressed before they escalate into costly system failures. In addition, real-time monitoring and control can also provide insight into system performance, enabling the optimisation of system parameters for increased efficiency and effectiveness.

Real-time monitoring and control are applicable to a wide range of systems, including manufacturing, transportation, and energy systems. In manufacturing, real-time monitoring and control can be used to optimise production processes, reducing waste and increasing throughput. In transportation, real-time monitoring and control can be used to optimise vehicle performance, reducing fuel consumption and improving safety. In energy systems, real-time monitoring and control can be used to optimise power generation and distribution, reducing costs and increasing reliability.

Zhou et al. [63] discussed the importance of condition monitoring (CM) in improving the reliability of rotating machinery (RM). They emphasised the need for an efficient CM method with simple and intuitive attributes for industrial applications. The development of health indicators (HIs) that connect fault detection, degradation assessment, and prognosis applications is crucial in CM. The paper reviews the construction methods of HIs for rotating machinery, covering both classical technical approaches and recent data-oriented intelligent methods such as deep learning. The benefits and potential of efficient HIs for condition monitoring are analysed, along with current challenges and future research opportunities.

By monitoring system parameters in real time, safety-critical faults can be detected and addressed before they pose a threat to system operators or the public. In addition, real-time monitoring and control can be used to implement safety protocols, such as emergency shutdown procedures, in the event of a system failure.

Overall, real-time monitoring and control are critical aspects of complex systems. By leveraging sensor data and advanced data analytics techniques, real-time monitoring and control can provide insight into system health and performance, enabling the optimisation of system parameters for increased efficiency and effectiveness. Real-time monitoring and control also have implications for system safety, enabling the detection and prevention of safety-critical faults.

### 6.2. Maintenance Optimisation

Maintenance optimisation is an essential aspect of complex systems, and it involves the use of various techniques to determine the optimal maintenance schedule for a system. The main goal of maintenance optimisation is to minimise the cost of maintenance while ensuring that the system operates at its optimal level. Two of the most commonly used maintenance optimisation techniques in IVHM systems are condition-based maintenance and predictive maintenance.

There are several factors to consider when selecting the appropriate maintenance optimisation technique for an IVHM system. These include the cost of maintenance, the criticality of the system, the availability of replacement parts, and the system’s operating environment. Additionally, it is important to select a maintenance optimisation technique that is compatible with the sensors and other monitoring tools used in the system.

In safety-critical systems, such as industrial plants or aircraft, the prevention of failure while maintaining high availability is crucial. Advanced prognostic algorithms and sensing techniques are being developed for predictive maintenance to achieve reliable and accurate prediction. However, there is a lack of in-depth studies on evaluating sensing techniques based on their prediction performance and inspection scheduling. Park et al. [64] addressed the need to evaluate the cost-effectiveness of different sensors by considering their contribution to reducing unnecessary inspection or measurement costs while maintaining prognosis performance. The authors conducted simulations to analyse prediction performance under varying measurement intervals and different levels of noise during degradation. Additionally, they analysed real run-to-fail (RTF) datasets from two different sensors to design an optimal measurement system for predictive maintenance. The study provides insights into selecting sensors based on cost-effectiveness and resistance to noise in order to improve maintenance strategies.

Demetriou et al. [65] introduced the economic aspect as a new factor in sensor selection for the improved filtering of dynamical systems. The price of a single sensor, represented by high covariance values, is considered to incorporate the economic perspective into sensor optimisation for optimal filtering. Instead of relying on a single expensive and highly accurate sensor, the unit price and total price of a network of inexpensive noisy sensors are utilised as alternatives. The study presents algorithms for integrated sensor optimisation for both finite and infinite dimensional systems and provides examples to illustrate the effects of considering economic aspects in sensor selection.

Moradi et al. [52] addressed the challenging problem of performing and updating risk and reliability assessments for complex engineering systems (CES) with high frequency. The complexity of operational data and system complexity necessitate the use of novel data-driven methods such as DL and engineering knowledge. The authors propose a mathematical architecture for the operation condition and risk monitoring of CES, utilising a Bayesian network (BN) to model system and subsystem relations, adverse event scenarios, and subsystem-level information fusion. Bayesian DL models are trained for subsystem diagnostics based on condition monitoring data, and their outputs are integrated into the BN. The proposed architecture effectively addresses both data and systems complexity, providing system-level insights and the ability to incorporate human inputs and qualitative information. The effectiveness of the approach is demonstrated through a case study on a vapour recovery unit at an offshore oil production platform.

In conclusion, the maintenance optimisation of complex systems and the use of condition-based maintenance and predictive maintenance techniques can significantly improve the diagnostic reliability and performance of the system. The selection of the appropriate maintenance optimisation technique should be based on several factors, including the cost of maintenance, the criticality of the system, and the operating environment.

### 6.3. Fault Diagnosis and Prognosis

This section focuses on the techniques used for fault diagnosis and prognosis in complex systems. These techniques are essential for operation optimisation and system availability, as they allow for the detection and prediction of system faults before they can lead to system failure.

Gao et al. [66] focused on the growing need for the early detection and identification of abnormalities and faults in industrial systems to minimise performance degradation and ensure safety. The authors highlight the importance of real-time fault diagnosis and fault-tolerant control methods in achieving these objectives. They provide a comprehensive review of fault diagnosis approaches and their applications, focusing on both model-based and signal-based perspectives. The paper aims to offer an extensive overview of the advancements in this field, with particular emphasis on the results reported in the last decade.

Baraldi et al. [25] proposed a general method for extracting a health indicator to measure the degradation state and predict the future evolution of industrial components. The method combines feature extraction techniques, including empirical mode decomposition and auto-associative kernel regression, with a multi-objective binary differential evolution (BDE) algorithm for optimal feature selection. The optimisation objectives focus on desired characteristics of the health indicator, such as monotonicity, trendability, and prognosability. A case study on turbofan engines is conducted to predict the remaining useful life. The results demonstrate the effectiveness of the proposed method in extracting accurate health indicators for prognostics.

### 6.4. Performance Optimisation

Performance optimisation is essential for efficient and effective IVHM, and sensor optimisation plays a necessary role in achieving this goal. IVHM systems need to be designed to optimise system performance, including operational efficiency, diagnostic reliability, availability, and maintainability. Performance optimisation aims to achieve these objectives by continuously monitoring and analysing system performance data and taking corrective actions when necessary.

Koutroulis et al. [67] reviewed the challenge of constructing comprehensive health indicators (HIs) in prognostics and health management (PHM) using large amounts of condition monitoring data. The authors propose a novel anticausal-based framework with reduced model complexity to predict the cause from the effects of causal models, specifically designed for complex systems operating under time-varying conditions. Two heuristic methods, complexity estimation and Granger causality, are used to infer the causal models. The framework demonstrates strong generalisation capabilities and robust online predictions of HIs, outperforming existing deep learning architectures in terms of average root-mean-square error (RMSE) by nearly 65%. The validation and comparison of the framework are conducted on NASA’s N-CMAPSS dataset recorded from a commercial jet, further enhancing the CMAPSS simulation model.

Overall, performance optimisation techniques enable the continuous monitoring and analysis of system performance data, allowing for real-time corrective actions to be taken to maintain optimal system performance. In conclusion, this section provides a comprehensive overview of sensor optimisation for improved operation in industrial systems. It covers various aspects, including real-time monitoring and control, maintenance optimisation, decision support systems, fault diagnosis and prognosis, and performance optimisation. The insights and strategies presented in this section contribute to the development of efficient and reliable sensor operation techniques for industrial applications.

### 6.5. Cost Function for Operation Optimisation

This section emphasises the significance of the cost function in guiding decision-making during sensor operation. The cost function for sensor operation plays a central role in achieving reliable, energy-efficient, and optimised sensor performance while considering the associated costs. It allows engineers and decision-makers to strike a balance between system diagnostic reliability, energy consumption, maintenance requirements, and performance improvements while adhering to budget constraints.

A comprehensive cost function for sensor operation includes the following factors:

1. System Reliability: Evaluating the ability of sensors to perform their intended functions consistently and accurately over extended periods without failure or disruption, lifespan considerations. System reliability can be quantified by evaluating the probability of the system operating without failure over a given period. This can be measured using metrics such as mean time between failures (MTBF), mean time to repair (MTTR), or availability percentage.
2. Energy Efficiency: Assessing the energy consumption of sensors during operation to ensure optimal energy utilisation and reduce overall power consumption. Energy efficiency can be quantified by evaluating the energy consumption of the system in relation to the desired output or task. This can be measured based on metrics such as energy per unit of data processed, energy per unit of time, or energy efficiency ratings.
3. Maintenance Cost: Considering the costs associated with routine maintenance, sensor calibration, and periodic servicing to sustain the sensor’s operational efficiency. Maintenance costs can be quantified by evaluating the expenses associated with maintaining and servicing the sensors and related components. This can be measured in terms of monetary costs, time required for maintenance activities, or the frequency of maintenance interventions.
4. Performance Optimisation: Quantifying the degree to which sensor operation aligns with the system’s performance objectives, ensuring optimal utilisation of sensor data for decision-making. Performance optimisation can be quantified by evaluating the improvement in system performance achieved through optimisation efforts. This can be measured based on metrics specific to the system, such as throughput, accuracy, response time, error rates, or any other performance-related indicators.
5. These approaches provide a starting point for quantifying the objective functions in the operation part of the sensor optimisation process. Depending on the specific context and requirements of the complex engineering system, the actual quantification methods and metrics may vary.
6. Security can be quantified by evaluating the level of protection against unauthorised access, data breaches, or cyber threats. This can be measured based on metrics such as security vulnerability assessments, penetration testing results, or compliance with security standards.
7. Sensor Longevity can be quantified by evaluating the expected lifespan or operational duration of the sensors. This can be measured based on mean time between failures (MTBF), sensor degradation rates, or estimated lifetime usage.
8. Sensor Environmental Impact can be quantified by evaluating the ecological footprint or sustainability aspects associated with the production, usage, and disposal of the sensors. This can be measured based on metrics such as carbon footprint, material recyclability, or compliance with environmental regulations.

These approaches provide a starting point for quantifying the objective functions in the operation part of the sensor optimisation process. The first four factors are considered for the general cost function in the operation part; however, depending on the specific context and requirements of the complex engineering system, the actual quantification methods and metrics may vary.

Cost (f) = α × System Reliability + β × Energy Efficiency + γ × Maintenance Cost + δ × Performance Optimisation

Quantifying the cost function for sensor operation involves assigning appropriate weights to each cost component based on its relative importance within the specific complex engineering system. These weights are determined through comprehensive analysis, considering factors such as system requirements, maintenance schedules, energy budgets, and performance goals. By employing multi-objective optimisation techniques, the cost function can be effectively integrated with other objective functions, such as system reliability, energy efficiency, maintenance costs, and performance optimisation, to achieve an optimal sensor operation configuration.

By incorporating a well-defined cost function into the sensor operation optimisation process, complex engineering systems can achieve reliable and energy-efficient sensor performance, optimising the overall efficiency and longevity of the system.

## 7. Conclusions

In this comprehensive literature review, the various aspects of sensor optimisation in complex engineering systems are explored. The review encompassed sensor selection, placement, data processing, and operation, each of which plays a crucial role in enhancing system performance, reliability, and cost-effectiveness.

Throughout the review, the importance of cost functions as indispensable tools for guiding decision-making in each stage of sensor optimisation is highlighted. The cost functions allowed engineers and decision-makers to strike a balance between performance requirements and financial considerations, ultimately leading to cost-effective and efficient sensor configurations.

In the sensor-selection stage (Section 3), how the cost function played a pivotal role in evaluating the economic implications of sensor choices is discussed. By considering acquisition costs, installation expenses, maintenance requirements, and other relevant factors, the cost function enabled the selection of sensors that aligned with project budgets while meeting system performance objectives.

Similarly, in the sensor placement stage (Section 4), the cost function facilitated optimal sensor deployment by accounting for coverage, connectivity, interference minimisation, and resource utilisation costs. Through comprehensive analysis, engineers achieved sensor placements that maximised system performance while minimising operational costs.

In data processing (Section 5), the cost function played a vital role in ensuring the efficient analysis and utilisation of sensor data. By balancing data accuracy, computational efficiency, feature relevance, and model complexity, the cost function enabled the extraction of valuable insights while minimising computational overhead and resource utilisation.

In sensor operation (Section 6), the cost function guided decisions to achieve reliable, energy-efficient, and cost-effective sensor performance. By considering system reliability, energy consumption, maintenance costs, and performance optimisation, the cost function optimised sensor operations to support the system’s long-term sustainability and performance excellence.

In conclusion, this literature review demonstrated the significant role that cost functions play in the optimisation of sensors in complex engineering systems. By considering cost implications alongside other performance metrics, cost functions enable well-informed decisions that lead to the success and sustainability of the system. As sensor technology continues to advance, the development and refinement of cost functions will remain essential in driving innovation and efficiency in the field of sensor optimisation.

### 7.1. Summary of Key Findings

Throughout this literature review on sensor optimisation in complex engineering systems, several key findings have emerged, shedding light on the crucial role of cost functions and their impact on decision-making. The review explored sensor selection, placement, data processing, and operation, along with an integrated approach for multi-objective sensor optimisation. In this section, the main findings that contribute to a deeper understanding of the significance of cost functions in sensor optimisation are summarised:

Balancing Performance and Cost: Cost functions provide a systematic approach to balancing sensor performance requirements with associated costs. By quantifying and weighing cost components, engineers and decision-makers can make informed choices that optimise system performance while adhering to budgetary constraints.

Cost-Effectiveness in Sensor Selection: The cost function for sensor selection considers various cost-related factors, such as sensor acquisition costs, installation expenses, maintenance, and long-term operational costs. By evaluating these components, decision-makers can select sensors that meet performance criteria while remaining cost-effective.

Optimising Sensor Placement Efficiency: In the context of sensor placement, the cost function guides decisions to achieve optimal sensor coverage, connectivity, interference minimisation, and resource utilisation. By incorporating cost considerations, engineers achieve cost-efficient sensor deployments without compromising system performance.

Efficient Data Processing Strategies: The cost function for data processing ensures the efficient utilisation of sensor data. By considering data accuracy, computational efficiency, feature relevance, and model complexity, engineers can extract valuable insights while minimising computational overhead and resource consumption.

Reliable and Energy-Efficient Sensor Operation: Cost functions play a vital role in optimising sensor operation, balancing system reliability, energy efficiency, maintenance costs, and performance improvements. By quantifying these cost components, decision-makers achieve reliable and energy-efficient sensor performance.

Integrated Multi-Objective Optimisation: The integrated approach to multi-objective sensor optimisation employs cost functions alongside other objective functions, resulting in balanced and efficient sensor configurations. Trade-off analysis and Pareto front analysis enable decision-makers to make informed choices that optimise multiple objectives simultaneously.

Role of Cost Functions in Future Sensor Optimisation: As technology evolves and sensor optimisation advances, cost functions will continue to be critical tools in guiding decision-making. By considering cost implications alongside other performance metrics, cost functions support innovation and efficiency in sensor optimisation.

In conclusion, this literature review underscores the vital role of cost functions in sensor optimisation for complex engineering systems. By effectively incorporating cost considerations throughout the sensor optimisation process, engineers and decision-makers can achieve cost-effective, efficient, and reliable sensor configurations. As the field of sensor technology continues to evolve, cost functions will remain indispensable in driving advancements and ensuring the successful implementation of optimised sensor solutions in diverse engineering applications.

### 7.2. Contributions and Implications

In this section, we will outline the contributions of this literature review on sensor optimisation and highlight its implications for research, engineering practice, and decision-making in complex engineering systems.

#### 7.2.1. Contributions

This literature review makes several key contributions to the understanding of sensor optimisation in complex engineering systems:

Comprehensive Overview: The review provides a comprehensive overview of sensor optimisation, covering essential aspects such as sensor selection, placement, data processing, and operation. By examining each stage of the optimisation process, the review offers a holistic perspective on the challenges and opportunities in achieving efficient and cost-effective sensor configurations. A taxonomy and a concept map of the area have been generated.

Role of Cost Functions: The review emphasises the critical role of cost functions in guiding decision-making at each stage of sensor optimisation. By quantifying the trade-offs between performance and cost, cost functions empower engineers and decision-makers to make informed choices that align with budgetary constraints while optimising system performance.

Integration of Objective Functions: The integrated approach to multi-objective sensor optimisation highlighted in the review illustrates the importance of considering all objective functions simultaneously. By combining cost functions with other performance metrics, decision-makers can achieve balanced and efficient sensor configurations that cater to multiple optimisation goals.

#### 7.2.2. Implications

The findings of this literature review have several implications for research, engineering practice, and decision-making in the field of sensor optimisation:

Informed Decision-Making: By incorporating cost functions into the optimisation process, decision-makers can make informed choices that balance system performance requirements with financial considerations. Cost functions provide a quantitative basis for decision-making, promoting cost-effective and efficient sensor configurations.

Performance-Driven Optimisation: The integration of cost functions with other objective functions emphasises the need for performance-driven optimisation. Decision-makers must consider not only cost implications but also overall system performance to achieve successful sensor configurations.

Future Research Directions: The review identifies emerging trends and future research directions, such as advancements in sensor technology and the integration of AI and machine learning. Researchers can use this information as a foundation to explore innovative approaches to sensor optimisation.

Based on the insights gained from this review, decision-makers involved in sensor optimisation in complex engineering systems are recommended to:

Integrate Cost Functions: Incorporate cost functions into the decision-making process at each stage of sensor optimisation. By quantifying cost implications, decision-makers can make cost-effective choices that align with system performance objectives.

Consider Multi-Objective Optimisation: Embrace an integrated approach to multi-objective optimisation, combining cost functions with other performance metrics. This ensures a balanced and efficient sensor configuration that meets multiple optimisation goals.

Monitor Emerging Trends: Stay informed about emerging trends and advancements in sensor technology. Continuously evaluate how these advancements can improve sensor optimisation practices and enhance system performance.

In conclusion, this literature review highlights the significant role of cost functions in guiding efficient and cost-effective sensor optimisation in complex engineering systems. By considering cost implications alongside other performance metrics, engineers and decision-makers can achieve optimised sensor configurations that support the success and sustainability of complex engineering applications. The review’s findings offer valuable insights for researchers, engineers, and decision-makers seeking to enhance sensor optimisation practices and improve decision-making in diverse engineering scenarios.

### 7.3. Recommendations for Future Research

This literature review has provided valuable insights into the role of cost functions and their impact on decision-making. Building on these findings, several areas for future research are recommended to further advance the field of sensor optimisation:

Enhancing Cost-Function Models: Future research can focus on refining and expanding cost-function models for sensor optimisation. This includes investigating advanced techniques for quantifying cost components and exploring novel approaches to weigh the importance of different cost factors in specific engineering applications.

Dynamic Cost Functions: Investigate the development of dynamic cost functions that adapt to changing operational conditions and evolving project budgets. Dynamic cost functions can provide real-time decision support, enabling sensor configurations that respond to varying performance requirements and cost constraints.

Uncertainty and Risk Analysis: Incorporate uncertainty and risk analysis into cost functions to account for uncertainties associated with sensor performance, cost estimations, and environmental variations. Understanding the impact of uncertainties on optimisation outcomes can lead to more robust and reliable sensor configurations.

Integration of Lifecycle Cost Analysis: Extend cost functions to include lifecycle cost analysis, considering the long-term costs associated with sensor maintenance, calibration, and replacement. Lifecycle cost analysis can provide a comprehensive view of cost implications over the sensor’s entire operational lifespan.

Optimisation Algorithms: Explore advanced optimisation algorithms that effectively handle the multi-objective nature of sensor optimisation. Investigate the application of evolutionary algorithms, genetic algorithms, and machine learning techniques to efficiently search for optimal sensor configurations within the multi-dimensional cost-performance space.

Real-World Deployment Studies: Conduct real-world deployment studies to validate the effectiveness of cost-function-driven sensor optimisation in practical engineering applications. These studies can provide valuable insights into the challenges and benefits of implementing cost-optimised sensor configurations in diverse industrial settings.

Benchmarking and Comparative Studies: Conduct benchmarking and comparative studies to evaluate the performance of different cost-function approaches and optimisation algorithms. Comparative studies can provide valuable insights into the strengths and limitations of various optimisation strategies and guide future research directions.

Industry–University Partnerships: Foster partnerships between academia and industry to bridge the gap between theoretical research and practical implementation. Collaborative projects can accelerate the adoption of cost-function-driven sensor optimisation in real-world engineering applications.

In the end, these recommendations offer a roadmap for future research endeavours in sensor optimisation, emphasising the continued advancement of cost-function models, multi-objective optimisation techniques, and real-world deployment studies. By addressing these research areas, the field of sensor optimisation can further evolve, enabling engineers and decision-makers to make informed choices that balance system performance and cost-effectiveness in complex engineering systems.

## Figures and Tables

**Table 1:** Search Results in Scopus Database.

TITLE–ABSTRACT–KEYWORDS	Document Results
(Sensor AND Optimisation)	75.169
(Sensor AND Optimisation) AND (Complex AND Systems)	10.103
(Sensor AND Optimisation) AND (Complex AND Systems) AND (aircraft)	602
(Sensor AND Optimisation) AND (Complex AND Systems) AND (aircraft) AND (diagnostic)	85
“Sensor Optimisation”	509
“Sensor Optimisation” AND “Complex Systems” OR “aircraft”	63
“Sensor Optimisation” AND “Complex Systems”	9

**Table 2:** Sensor-Selection Techniques Encountered and the Associated References.

Researcher	Technique	Application
Kulkarni et al. [2]	Proposes a method that utilises a scalable multi-objective framework for sensor selection to maximise fault detection rate while minimising the total cost of sensors	A wind turbine gearbox is considered to demonstrate the efficacy of the proposed framework.
Santi et al. [3]	A model-based procedure (S4) that systematically selects an optimal sensor suite for overall health assessment of a designated host system.	In-space propulsion health management systems
Maul et al. [4]	S4 selected as the framework for further development and verification. Segmented into three groups: Knowledge Base, Iterative Down-Select Process, and Final Selection.	Applied to subsystem components of the Space Shuttle Main Engine
K. Nakai et al. [7]	Objective functions based on D-, A-, and E-optimality criteria of optimal design are adopted to greedy methods.	Applied to randomly generated systems and a practical dataset concerning the global climate.
Guan et al. [8]	Proposes a comprehensive evaluation method of sensor selection for PHM based on grey clustering.	Illustrated by an electronic control system, in which the effectiveness of different methods compared
Joshi et al. [9]	Convex Optimisation	The problem of choosing sensors or measurements, from among a set of candidate measurements, to obtain the best resulting estimate of some parameters
Shamaiah et al. [10]	Greedy Sensor-selection Algorithm	The problem of sensor selection in resource constrained sensor networks.
Wang et al. [11]	Entropy-based sensor-selection method which can provide quantitative description of the information contained in sensor data	Condition monitoring and prognostics of aircraft engine
Xu et al. [12]	Multi-objective Genetic Algorithm	Aircraft Engines
Najjar et al. [13]	The minimum Redundancy Maximum Relevance (mRMR) criterion with unsupervised embedded algorithm	Heat Exchanger Fouling Diagnosis in Aerospace Systems
Jiao et al. [14]	Improved Binary Wolf Pack Algorithm	Typical discrete combinatorial optimisation problem
Manohar et al. [15]	Balanced Model Reduction	Closed-loop Flow Control
Yan et al. [16]	Hybrid Bayesian fisher information and mutual information	Unreliable sensor networks

**Table 3:** Comparison of the Optimisation Techniques.

Optimisation Technique	Pros	Cons
Statistical Analysis	Provides valuable insights from data analysis	May require large datasets and complex statistical methods
Mathematical Modelling	Enables accurate representation of system behaviour	Can be computationally intensive for complex systems
Simulation Techniques	Allows testing in virtual environments	May not fully capture real-world complexities
Inverse Problems	Determines source location from sensor measurements	Can be sensitive to measurement errors and noise
Gaussian Process (GP)	Accounts for uncertainty in predictions	May require significant computational resources

**Table 4:** Comparison of the Optimisation Approaches.

Approach	Pros	Cons
D-, A-, E-optimality	Evaluates optimality based on specific criteria	May not consider other important aspects of sensor placement
Greedy Algorithms	Relatively simple to implement	May not always find the global optimum

**Table 5:** Comparison of the Optimisation Approaches.

Approach	Pros	Cons
Singular Value Decomposition	Useful for large-scale structures	May not be applicable to all types of systems
QR Decomposition	Efficient for sensor placement	Limited to certain types of optimisation problems
Fuzzy Measurement Coverage	Incorporates uncertainties in coverage	Requires careful tuning of fuzzy parameters

**Table 6:** Comparison of the Optimisation Approaches.

Approach	Pros	Cons
Mutual Information	Exploits sub-modularity for efficiency	NP-complete problem, may not find the global optimum
Lazy Evaluations	Speeds up optimisation process	May not fully capture all system dynamics
Robust Placements	Considers uncertainties and node failures	Requires additional computational complexity

**Table 7:** Comparison of the Placement Optimisation Methods.

Methods	Pros	Cons
Dynamic Fault Tree	Captures time-varying dependencies	Complexity increases with system size
Firefly Algorithm (FA)	Efficient for large-scale structures	May require parameter tuning
Dynamic Bayesian Network	Accounts for time dependencies	Requires accurate model representation
Genetic Algorithms	Global search capability	Convergence may be slow
Particle Swarm Optimisation (PSO)	Converges quickly	May get stuck in local optima
Sensitivity-Based Approaches	Identifies sensitive locations	Sensitive to measurement errors
Topology-Based Approaches	Identifies critical locations	Complexity increases with system size

## Data Availability

Data are available on request.
